# Faculty perceptions and knowledge of career development of trainees in biomedical science: What do we (think we) know?

**DOI:** 10.1371/journal.pone.0210189

**Published:** 2019-01-30

**Authors:** Stephanie W. Watts, Deepshikha Chatterjee, Julie W. Rojewski, Carol Shoshkes Reiss, Tracey Baas, Kathleen L. Gould, Abigail M. Brown, Roger Chalkley, Patrick Brandt, Inge Wefes, Linda Hyman, J. Kevin Ford

**Affiliations:** 1 Michigan State University, East Lansing, MI, United States of America; 2 New York University, New York, NY, United States of America; 3 University of Rochester Medical Center, Rochester, NY, United States of America; 4 Vanderbilt University School of Medicine, Nashville, TN, United States of America; 5 University of North Carolina, Chapel Hill, NC, United States of America; 6 University of Colorado Denver Anschutz Medical Campus, Denver, CO, United States of America; 7 Boston University School of Medicine, Boston, MA, United States of America; Universidade de Mogi das Cruzes, BRAZIL

## Abstract

The **B**roadening **E**xperiences in **S**cientific **T**raining (BEST) program is an NIH-funded effort testing the impact of career development interventions (*e*.*g*. internships, workshops, classes) on biomedical trainees (graduate students and postdoctoral fellows). BEST Programs seek to increase trainees’ knowledge, skills and confidence to explore and pursue expanded career options, as well as to increase training in new skills that enable multiple career pathways. Faculty mentors are vital to a trainee’s professional development, but data about how faculty members of biomedical trainees view the value of, and the time spent on, career development are lacking. Seven BEST institutions investigated this issue by conducting faculty surveys during their BEST experiment. The survey intent was to understand faculty perceptions around professional and career development for their trainees. Two different, complementary surveys were employed, one designed by Michigan State University (MSU) and the other by Vanderbilt University. Faculty (592) across five institutions responded to the MSU survey; 225 faculty members from two institutions responded to the Vanderbilt University survey. Participating faculty were largely tenure track and male; approximately 1/3 had spent time in a professional position outside of academia. Respondents felt a sense of urgency in introducing broad career activities for trainees given a recognized shortage of tenure track positions. They reported believing career development needs are different between a graduate student and postdoctoral fellow, and they indicated that they actively mentor trainees in career development. However, faculty were uncertain as to whether they actually have the knowledge or training to do so effectively. Faculty perceived that trainees themselves lack a knowledge base of skills that are of interest to non-academic employers. Thus, there is a need for exposure and training in such skills. Faculty stated unequivocally that institutional support for career development is important and needed. BEST Programs were considered beneficial to trainees, but the awareness of local BEST Programs and the national BEST Consortium was low at the time surveys were employed at some institutions. It is our hope that the work presented here will increase the awareness of the BEST national effort and the need for further career development for biomedical trainees.

## Introduction

In 2012, the NIH issued a workforce report on biomedical trainees and their future careers [1]. This followed a *Science* paper urging greater emphasis on career training for all graduate students [2]. On the basis of such reports, the NIH launched the BEST (Broadening Experiences in Scientific Training) initiative through a funding opportunity supported by the Director’s Biomedical Research Workforce Innovative Award Program [3]. The call for proposals stated that “despite the broad range of career options available to U.S.-trained Ph.D. biomedical scientists, graduate programs and postdoctoral training focus almost exclusively on preparing individuals for careers as academic researchers. The committee recommended that NIH-supported graduate programs and post-doctoral awareness be broadened to reflect the actual career outcomes of today’s PhD graduates and postdoctoral scientists” [3]. This effort to expand professional development training in biomedical careers is supported by findings that “scientists from all social backgrounds showed significantly decreased interest in faculty careers at research universities, and significantly increased interest in non-research careers at PhD completion relative to entry” [4]. Similarly, research on biomedical trainees supported that students were actively considering and exploring a broad range of careers [5].

The NIH-issued call for proposals asked for the creation of innovative approaches which would be implemented and rigorously tested. NIH awarded BEST grants to 17 different institutions: 10 in 2013 and 7 in 2014. Collectively, they work together through the NIH BEST Consortium (www.nihbest.org). This group of 17 institutions convenes monthly over a webinar, and yearly in a national meeting to share ideas and resources so as to leverage research projects, and serve as a hub of information and expertise for other groups interested in exploring career development for biomedical trainees. Institutions have been tracking how interventions such as workshops, experiential learning opportunities, co-curricular learning opportunities, and other strategies most effectively help a biomedical trainee find/establish a career in different biomedical sectors, not just in academia. All BEST institutions have participated in rigorous evaluation and analysis. After at least four years of collecting data, the BEST Consortium is analyzing and publicizing findings about the impact of these awards. This paper stands as an example of such an effort.

Each institution entered into the BEST experiment knowing that faculty mentors would be critical partners in this experiment. The success or failure of BEST efforts relies upon their trainees as subjects in the BEST experiment. Though the impact of BEST initiatives on *faculty members* was not a focus of any particular award effort, BEST institutions were cognizant that having faculty buy-in to this experiment was essential. It was important that faculty mentors saw value in their trainees participation in professional development opportunities and supported their trainees in using an appropriate time for such training.

As a group, we recognized that capturing data about faculty perception was vital. This included examining faculty attitudes about the value of having BEST trainees spend time “outside the lab” to complement their traditional scientific training, querying who most faculty believed should provide such training, and ultimately, asking how faculty saw the intersections between scholarly and scientific training, and the professionally-oriented efforts of BEST programs.

Entering into the BEST experiment, there was the question of whether faculty were resistant to broadening career options for trainees beyond that of traditional academic faculty positions. However, empirical analysis was lacking on how faculty view broadening career options in STEM. To address these gaps in knowledge, both Vanderbilt University and Michigan State University independently developed a survey to administer to biomedical faculty. Though there are some differences in the surveys themselves (see Appendix 1 and Appendix 2), both were designed to gauge perceptions from faculty members about their knowledge of and attitudes about career development for biomedical trainees. These two surveys were created and administered independently from one another. Both Vanderbilt and MSU offered their survey instrument to other BEST institutions to use. Seven total institutions participated. Two offered the Vanderbilt Survey, and five administered the MSU Survey at their institution. We report the findings in this way to recognize and preserve differences between the two surveys, but share the findings from both in this single publication because their shared dimensions provide other useful insight and affirmation of findings.

## Methods

### Creation of two independent surveys

#### MSU

The MSU BEST survey team consisted of experts from industrial and organizational psychology, education, and biomedical science. Together, they designed a Qualtrics survey that was shared among any BEST institutions who wished to capture faculty views on professional development for their trainees in this manner. In this survey, faculty were asked to consider graduate students and postdoctoral fellows as an aggregate when answering questions, except for a few questions that addressed these two groups individually. Five institutions administered the MSU survey. In the rest of this report, for ease of reporting, we refer to the “MSU Survey” and include data submitted by all five institutions.

#### Vanderbilt

The Vanderbilt BEST team created a survey on Survey Monkey and also offered this instrument to any interested BEST campuses. In this survey, questions were, in places, more pointed in asking about training for post-doctoral scholars *vs* graduate students. Two institutions administered the Vanderbilt survey. In the rest of this report, we refer to the “Vanderbilt Survey” to include data from both institutions that administered this survey.

### Faculty surveyed

The surveys, both MSU and Vanderbilt, were sent to the faculty of department/programs that have a focus on biomedical sciences. The inclusion/exclusion of departments was at the discretion of the BEST team administering the survey at each institution. This included, for example, departments of biochemistry, genetics, pharmacology, physiology that have clear missions in biomedical departments as well as departments less obviously biomedical such as mechanical engineering (in which biomedical engineering is a part).

### Survey administration

Each Institution had its own IRB approval for activities supported by the BEST grant, and this work was done under the auspices of these approvals. These were as follows (institute, ethics committee/institution review board name, IRB approval number): MSU, Office of Regulatory Affiars, MSU Institutional Review Board, x13-824e; UNC, UNC Office of Human Research Ethics/Institutional Review Board, 14–0544; Boston, Boston University Medical Campus Institutional Review Board, H-33268; Rochester, Research Subjects Review Board, RSRB00055304; Vanderbilt, Institutional Review Board, Behavioral Sciences, 160600; Colorado, University of Colorado Institutional Review Board, 13–2349; and NYU, Office of Science and Research Institutional Review Board, i13-00727. All participants could voluntarily take this survey, and participation was considered consenting with the publication of anonymous data. In all cases, individuals were not identified by name. Recruiting strategies for taking surveys differed slightly at each campus. In general, the survey was introduced over e-mail, and faculty had three weeks to respond, with a weekly reminder sent to them.

Data were grouped within a survey such that no one institution can be identified. **Table 1** shares the dates when each campus administered its survey and notes at what point during each campus’s 5-year BEST grant effort the survey was administered.

**Table 1:** Characteristics of Institutions and BEST programs involved in faculty surveys.

Institution	Type of BEST Program	Start of BEST Program	Survey Administration
**Michigan State University**	Cohort only	September 2014	April-May 2017
**University of Rochester**		September 2014	June 2017
**New York University** **School of Medicine**		September 2013	December 2017
**Boston University**		September 2014	January 2018
**University of North Carolina** **Chapel Hill**	A la Carte	September 2014	February 2018
**Vanderbilt University**		September 2013	May 2016
**University of Colorado, Denver-Anschutz Campus**		September 2013	September 2016

A la carte = open to all; Cohort = restricted.

### Data collection

Each institution collected their data, either in Qualtrics (converted to Pivot tables in Excel; MSU Based Survey) or in Survey Monkey (VU Survey). Results from these surveys—in which data were all personally deidentified—were shared by each institution with the MSU BEST team with the mutual agreement that data would be merged, cleaned up for clarity and alignment, and then analyzed collectively for the purposes of a single analysis. Data generated within the MSU survey were grouped by factor based questions by each individual institution, and these were the data sent to MSU. The data from the VU survey came directly from each institution, as these questions were not grouped by factor. Data were maintained securely at the host campuses as well as at MSU, and all data were kept on password-protected computers in locked offices. All files received at MSU were completely de-identified, save for the name of the institution.

### Data analyses and presentation

#### MSU based survey (S1 File)

For Qualtrics based data, Excel pivot tables were used to convert qualitative answers (strongly agree, agree, neutral, disagree, strongly disagree; yes or no) into a numerical count and percentage of the total. MSU’s survey was built around factors/scales—a single theme that the research team was interested in testing. The survey questions were then written in such a way that individual questions (or items) pointed back to the predominant factor/scale. In this way, analysis was able to determine if respondents answered consistently among questions that were grouped within the same factor/scale. Between two and seven questions were posed that spoke to each main factor/scale. Each question/item, aside from demographic data, aligned with one of the factors/scale that were the focus of the study.

In analyzing individual questions and how well they aligned with each factor/scale, all questions had equal weight in arriving at a final value for that factor/scale. Each institution’s responses for a factor/scale was averaged with that of the other four institutions. The graphs shown represent those institutions (N = 5) that responded to the MSU survey poised as an average ± the standard error of the mean (SEM). The maximum number of respondents, collectively, was 592. A number reported on a graph that is less than 592 indicates that not all respondents answered this question. The average response rate for the MSU Survey was ~15% across institutions.

#### Vanderbilt based survey (S2 File)

Data collected from the two survey sites were averaged across institutions. This survey was not factor-based so each question was individually posed and analyzed separately. The graphs shown represent the mathematical average of the responses from those institutions (N = 2) that administered the Vanderbilt Survey. The maximum number of respondents, collectively, was 225. A number reported on a graph that is less than 225 indicates that not all respondents answered each question. Error bars are not present on any values from this specific survey because only two institutions took this particular survey. The average response rate for this survey was ~40% across institutions.

## Results

### Faculty demographics

**Table 1** shows information about the institutions in which the surveys were conducted, and the time during the BEST experiment in which the survey was administered.

**Fig 1:**
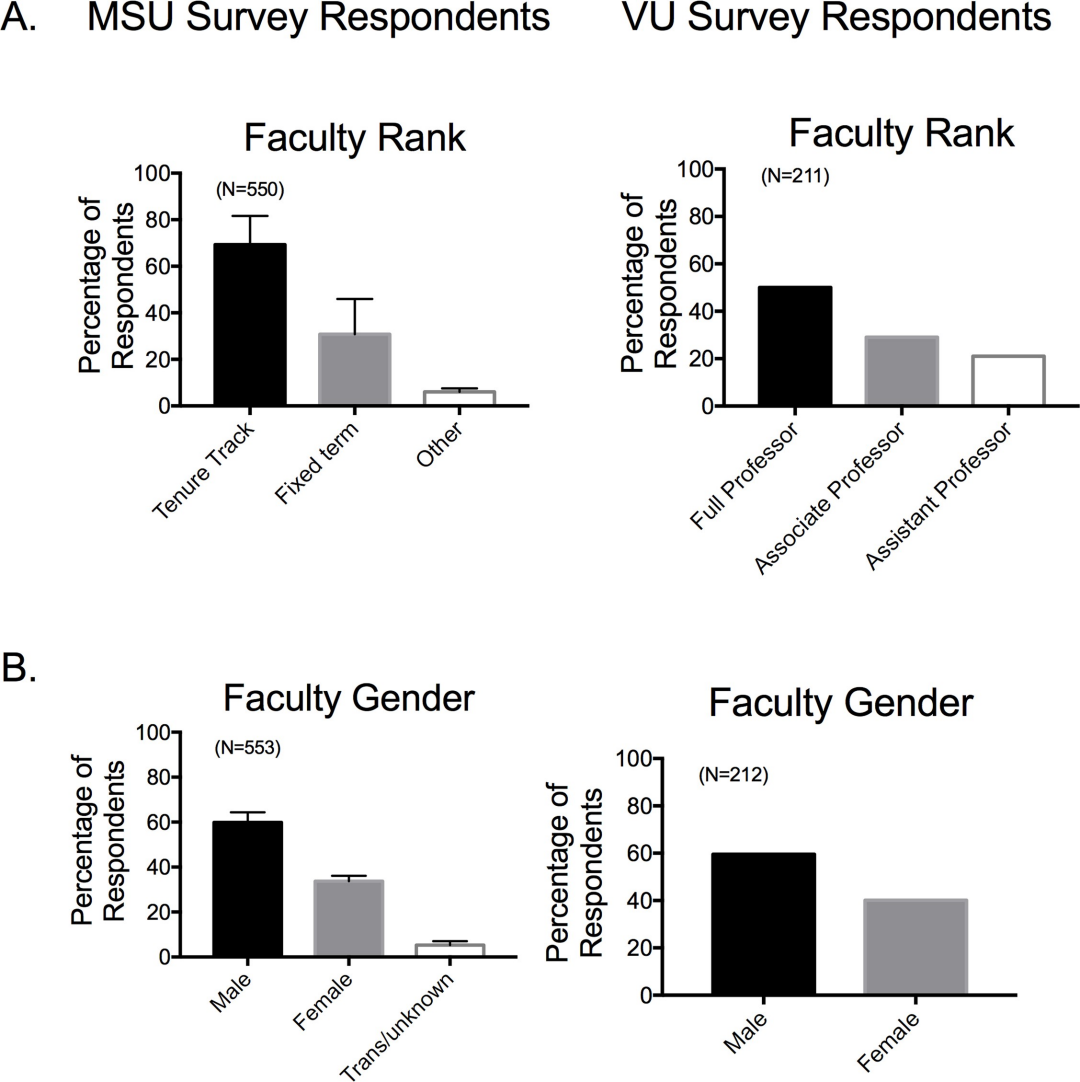
Demography (A rank; B gender) of faculty who took MSU (left) and VU (right) survey. Bars are means±SEM for number of faculty indicated in parentheses.

**Fig 1** shows the breakdown of faculty rank (**Fig 1A**) and gender (**Fig 1B**) for both surveys, all self-identified. Categories are different between the two institutions because this question was asked differently. For the MSU survey, the tenure-track category includes Full, Associate and Assistant professors while those self-identifying as fixed term are not tenure track. VU survey respondents were asked only about rank. Faculty from five institutions that participated in the MSU Survey were predominantly in tenure-track roles and male. For those that participated in the Vanderbilt Survey, they too were predominantly male and mid- to late-career, tenure-track faculty members (Full Professor, Associate Professor). The gender of the respondents reflects that of the faculty at large.

### Faculty history

**Fig 2:**
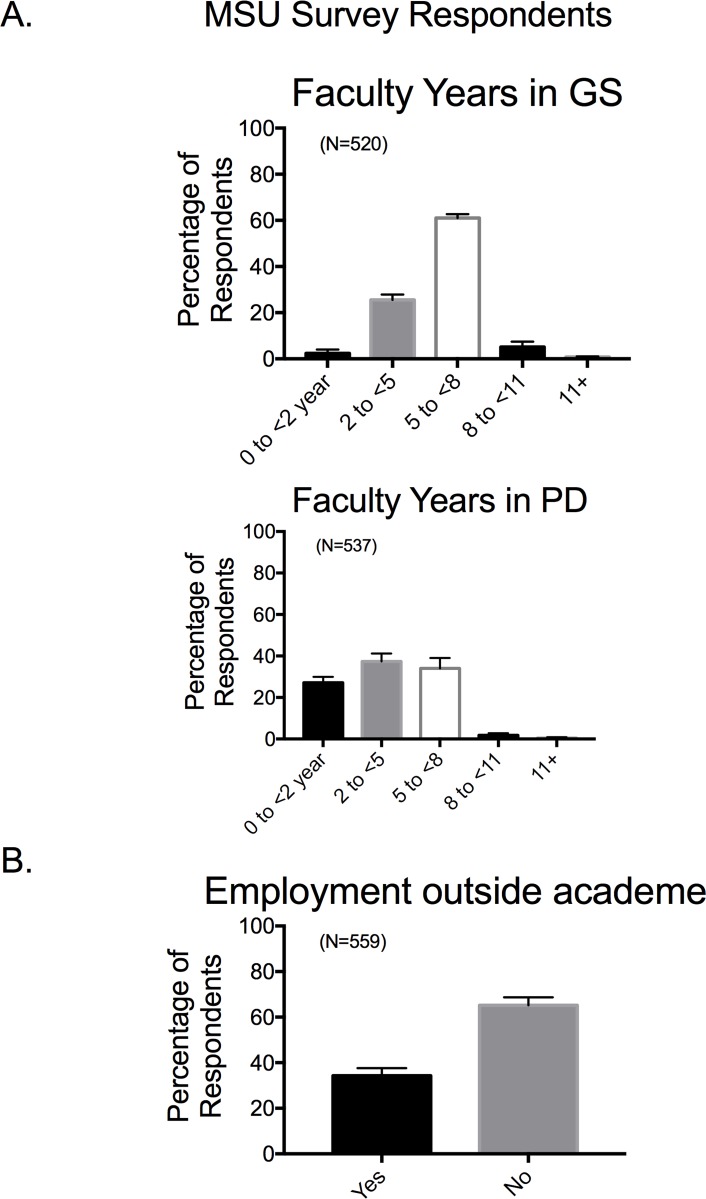
Faculty years spent in graduate training, postdoctoral training (A) or in employment outside academe (B). Bars are means±SEM for number of faculty indicated in parentheses. Reported for MSU based Survey respondents only.

In the MSU survey, faculty were asked how much time they spent on their own career development during their graduate and postdoctoral training. **Fig 2A** shows that a majority of faculty spent at least five years as a graduate student, with a few individuals spending as many as 11 years. This could include clinical training for those also training for an MD, DO or DVM degree. Time in postdoctoral fellowships was shorter, averaging from 2 to <8 years (**Fig 2A**). Some individuals responded that they did not have a postdoctoral fellowship, and they are included in the 0 to <2 years group. Interestingly, over 30% of the faculty who responded to these surveys had also worked outside of academia (**Fig 2B**), indicating that the academic world was not the only context in which they had worked, and thus had personal/professional experiences about other careers to share with their own trainees about other careers.

### Faculty training history

**Fig 3:**
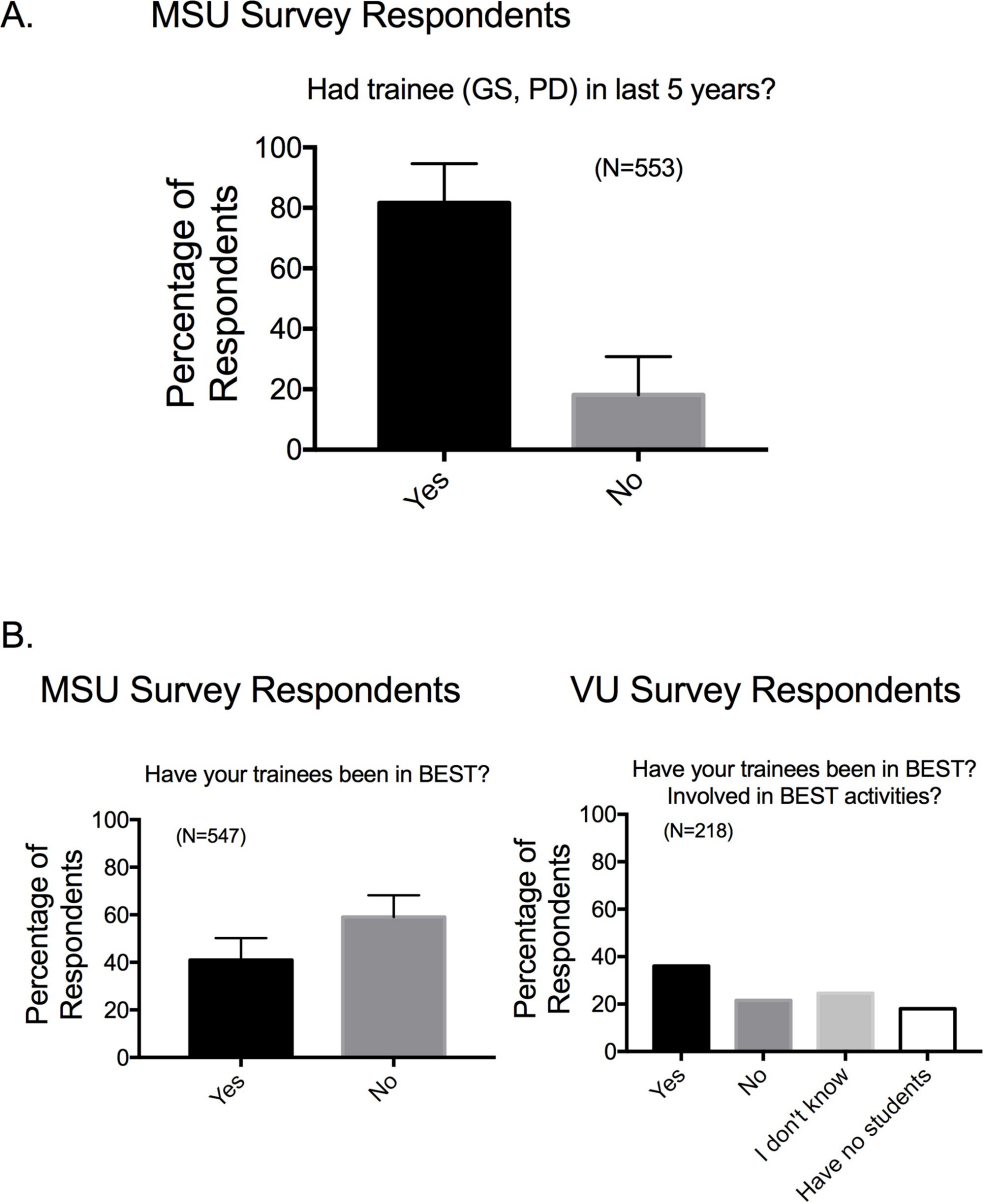
Response to whether faculty had had biomedical trainees (A) or BEST trainees (B). Bars are means±SEM for number of faculty indicated in parentheses.

To understand if survey respondents were directly involved in biomedical training, we asked whether 1) the faculty trained either a graduate student or postdoctoral fellow in the last five years (**Fig 3A**); and whether 2) the faculty had a trainee participate in BEST (**Fig 3B**). Most faculty had mentored a trainee in the last 5 years, but had not had a trainee who participated in a BEST program.

### Breakdown of factors for MSU survey

#### Reliability

The reliability of each factor/scale is expressed as an alpha value, where over 0.7 indicates an adequate reliability. Reliabilities were as follows: faculty knowledge base (0.65); student career knowledge base (0.69); mentoring (0.64); perceptions of departmental support (0.86); perceptions of colleague support (0.86); perceptions of faculty (PI) support (0.77); perceptions of graduate school support (0.79); and student benefits from BEST (0.88). Those items that were single measures have no reliability value.

### •Urgency

**Fig 4:**
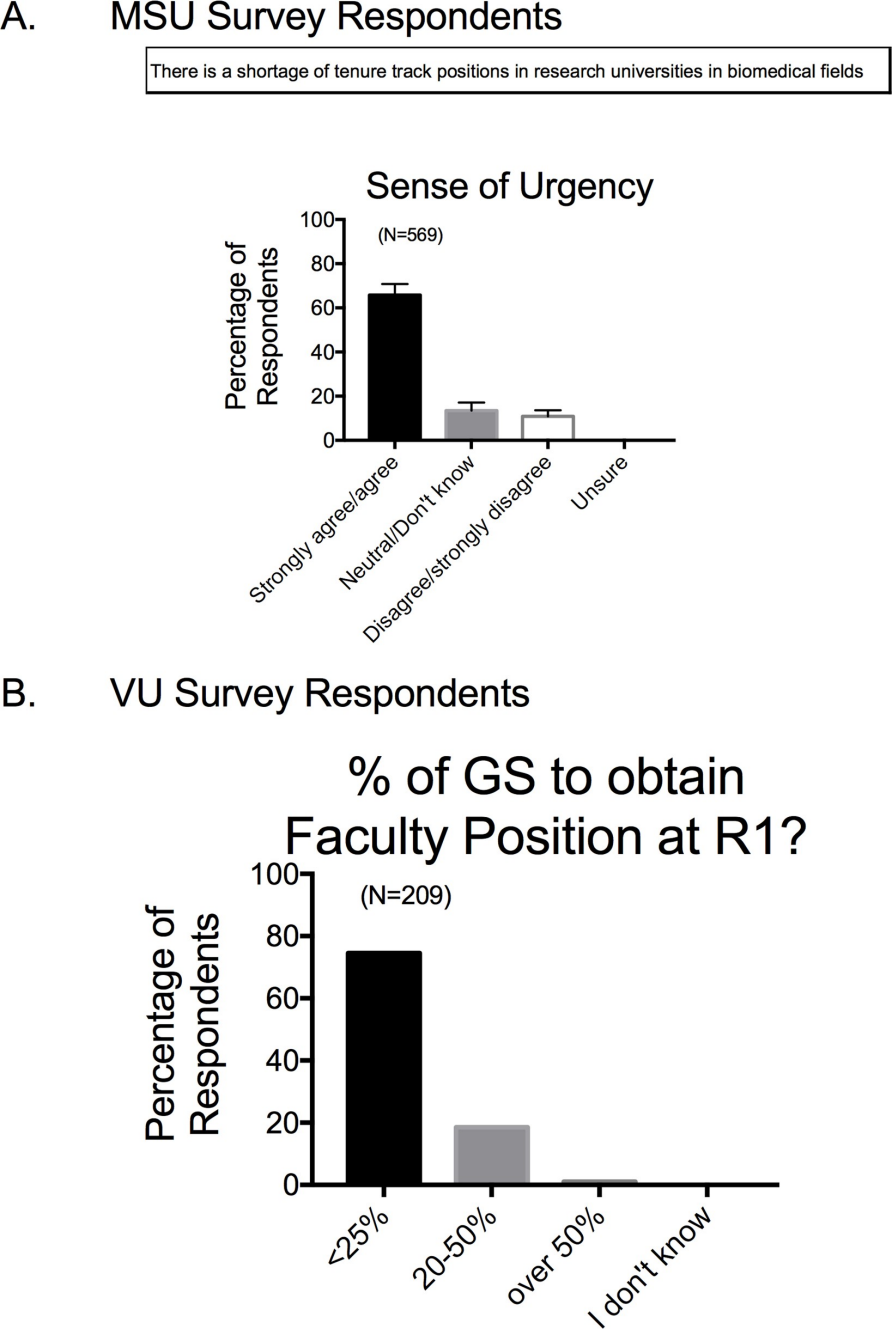
Responses to survey factor of Urgency for considering career development for MSU (A) and VU (B) respondents. Survey factor question for MSU based survey is shown in the top of panel A. Bars are means±SEM for number of faculty indicated in parentheses.

In the MSU Survey, faculty agreed that there is a shortage of tenure track positions in research universities in the biomedical fields (**Fig 4A**). The Vanderbilt survey posed this in a different way, asking whether faculty believed their trainees would be able to obtain a faculty position at an R1 institution. A majority stated that they believed less than 25% of those they trained would obtain such a position (**Fig 4B**). Collectively, these data support that faculty are aware that a majority of biomedical trainees are motivated towards a career other than an academic position. This aligns with the most recent data from the NIH which indicate that only approximately 23% of biomedical trainees will work in the tenure-track work force in academia.

### •Faculty mentoring, career knowledge, and individual support for trainees

**Fig 5:**
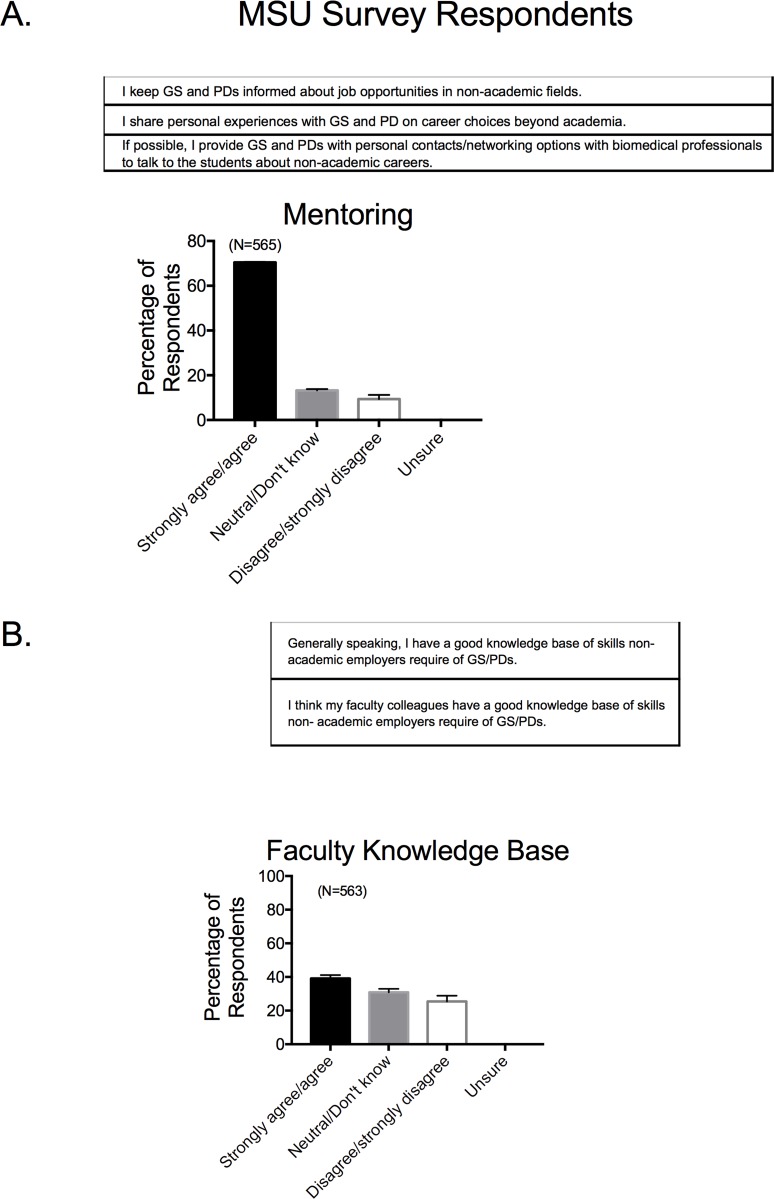
Response to survey factor of individual faculty mentoring perceptions (A) and career knowledge base (B). Survey factor questions for MSU based survey are shown in the top of each panel. Bars are means±SEM for number of faculty indicated in parentheses. Reported for MSU based Survey respondents only.

**Fig 6:**
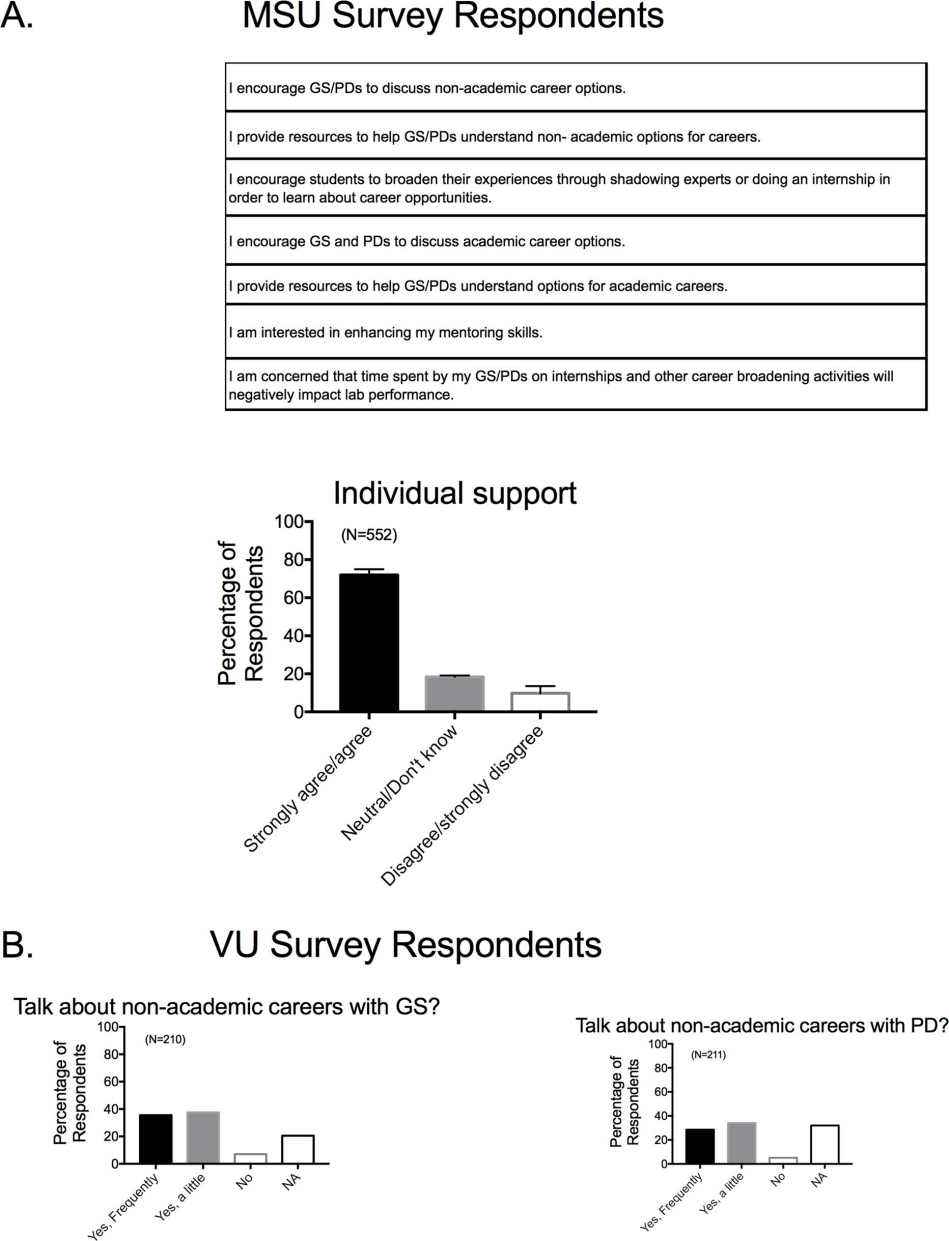
Response to survey factor of individual faculty career mentoring perceptions by MSU survey based faculty (A) and VU based faculty as divided by graduate students (left) and postdoctoral fellows (right; B). Survey factor questions for MSU based survey are shown in the top of the panel. Bars are means±SEM for number of faculty indicated in parentheses.

**Fig 7:**
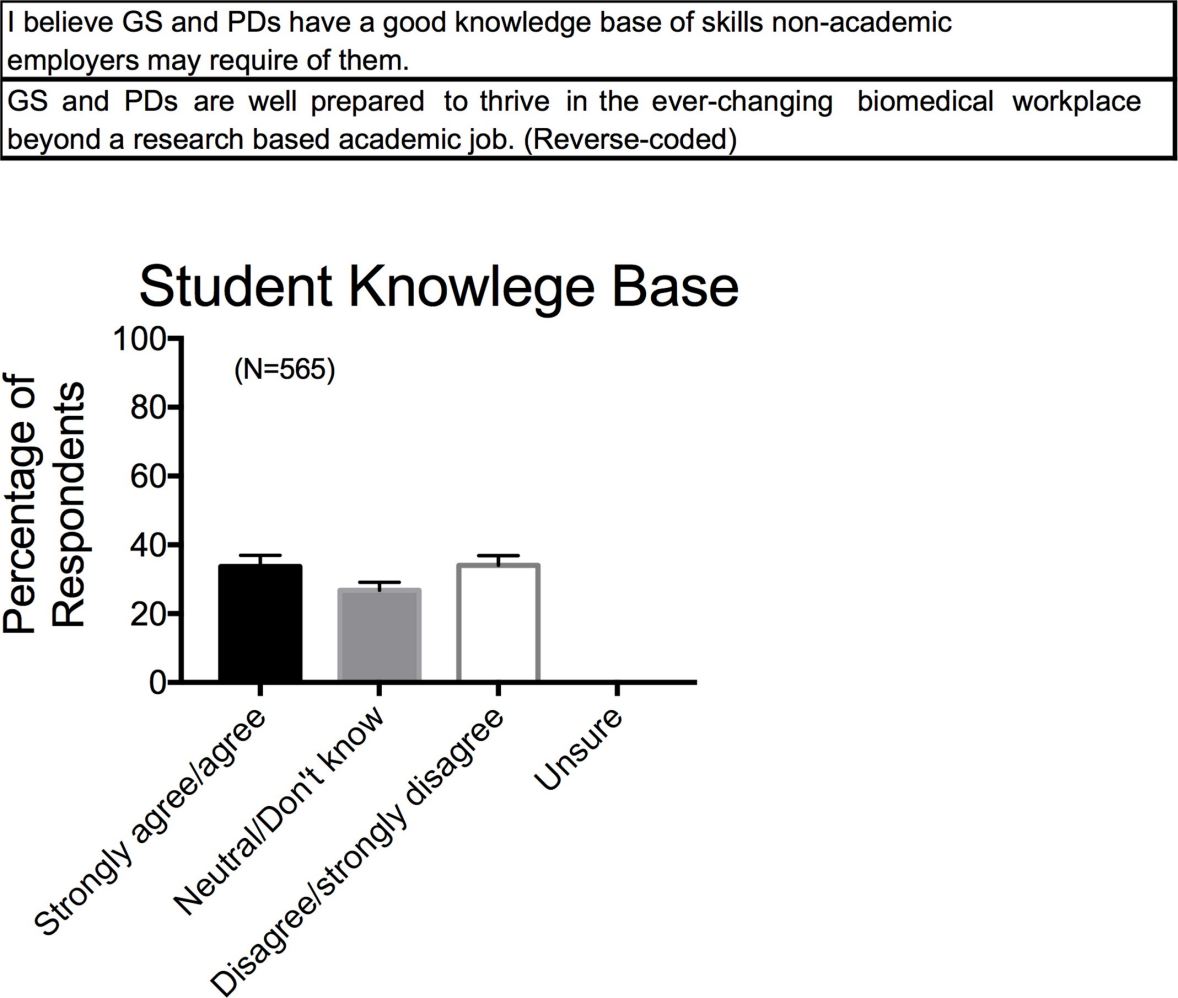
Response to survey factor of perceptions of trainee knowledge base of skills needed for non-academic employers and preparedness. Survey factor questions for MSU based survey are shown in the top of each panel. Bars are means±SEM for number of faculty indicated in parentheses. Reported for MSU based Survey respondents only.

Faculty state that they actively discuss non-academic career opportunities with trainees (**Fig 5A**) but a majority of faculty indicate that they themselves do not have a good knowledge of the skills needed in non-academic fields (**Fig 5B**). As such, there is a mismatch between the perceived urgency in career development training for biomedical scientists, and an individual faculty member’s ability to provide such training. Faculty report being engaged in supporting their trainees in considering career opportunities (**Fig 6A and 6B**), with a strong majority from all institutions stating they talk both to graduate students and postdoctoral fellows about non-academic careers. Even with this dedication, a majority of faculty believe that traditionally educated graduate students have an insufficient knowledge base of skills that non-academic employers require, and thus may be unprepared for the current biomedical work force (**Fig 7**). As such, traditionally educated trainees need more extensive training in skills that non-academic employers require, though these areas are outside the expertize fields of some faculty members. These perceptions solidify the need for BEST Programs which provide the resources that help trainees explore expanded career opportunities, and acknowledge the belief that mentors themselves are not fully equipped to offer this training.

### •Colleague and department support in career development

**Fig 8:**
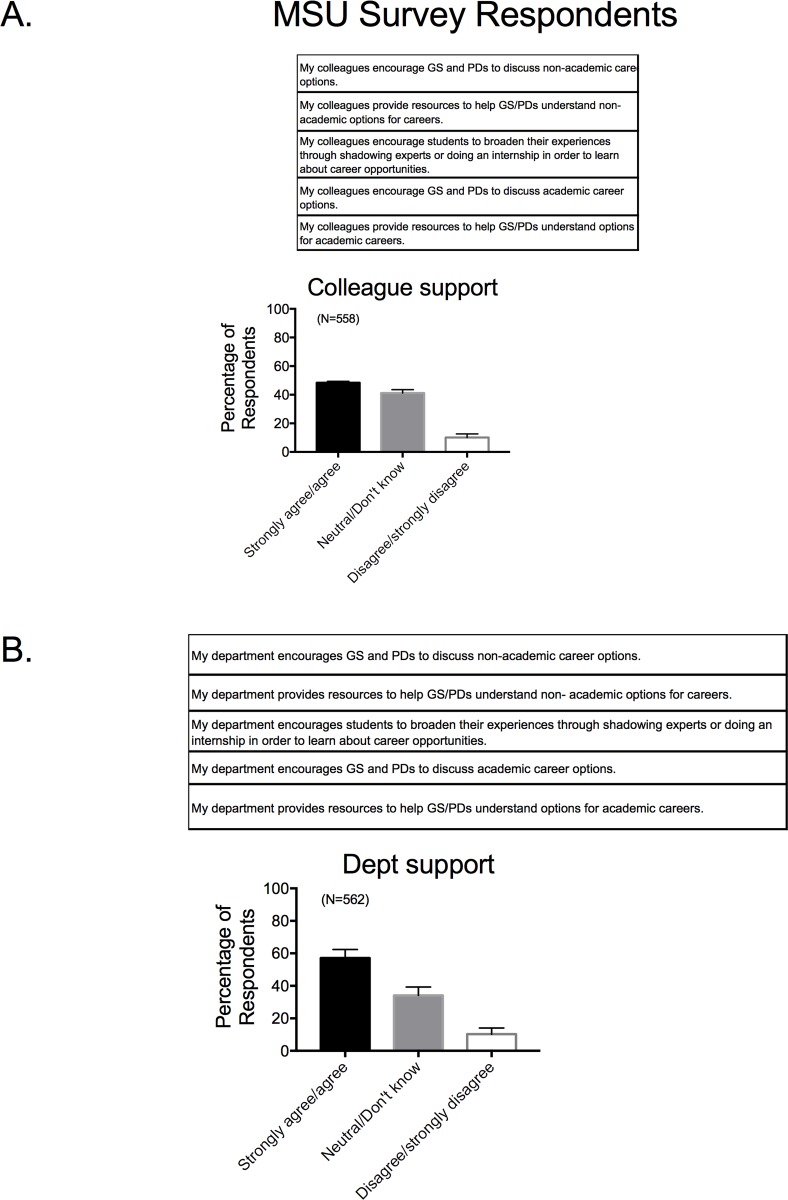
Response to survey factor of perceptions of colleague (A) and departmental (B) support in career development. Survey factor questions for MSU based survey are shown in the top of each panel. Bars are means±SEM for number of faculty indicated in parentheses. Reported for MSU based Survey respondents only.

The previous questions asked faculty to consider their individual knowledge surrounding career development of biomedical trainees. Similar questions were asked as to their knowledge/perceptions of what respondents believe happens among their colleagues, as well as what their department does for career development of biomedical trainees. We asked this question not only because we wanted to gauge perception about the departmental landscapes within which trainees train, but also to gauge faculty cultural norms around non-academic professional development. These questions were posed to get a sense of the climate of a department and collegial group relative to career development, acknowledging that climate change can only be fostered with purpose and intention. Despite generally reporting that they themselves (>70%) spoke to their trainees about a myriad of professional careers, faculty respondents reported being less sure (~50%) that their colleagues provided the same kind of discussion, resources, and opportunities that they themselves provided (**Fig 8A**). A similar trend was observed at the level of the department, but with a greater percentage of faculty believing a department does support trainee career development (**Fig 8B**). The micro-communities of laboratories within a department and the department itself are places that career development could take place.

### •BEST benefits to biomedical trainees

**Fig 9:**
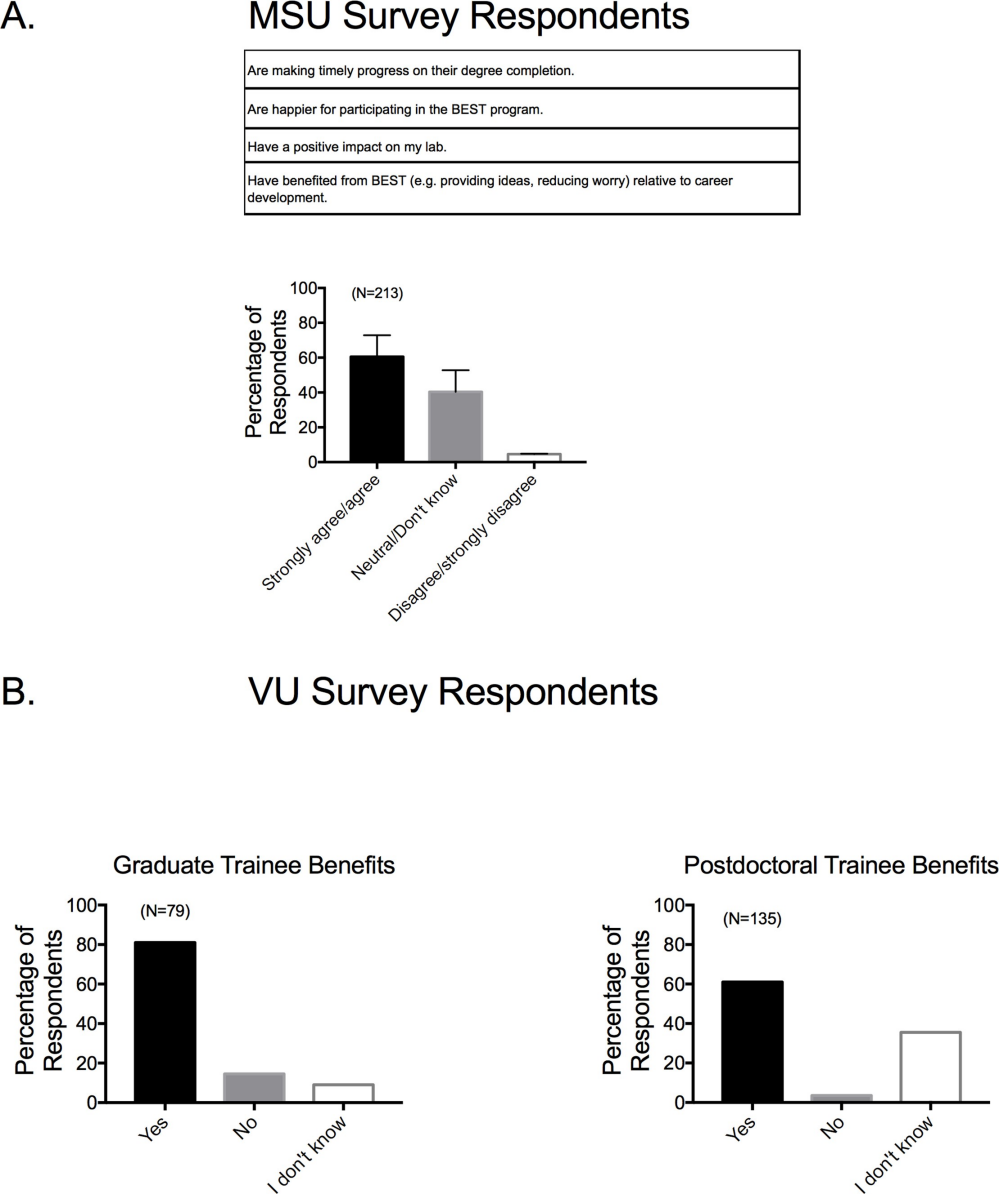
Responses to survey factor of perceived trainee benefits from BEST for MSU (A) and VU (B) respondents as divided by graduate students (left) and postdoctoral fellows (right). Survey factor questions for MSU based survey are shown in the top of the panel. Bars are means±SEM for number of faculty indicated in parentheses.

For those who were actively mentoring at least one trainee, faculty from the five campuses participating in the MSU Survey responded that they agreed that their trainees were making timely progress toward degree completion, were happier, had a positive impact on the lab and truly benefited from co-curricular, professional development programs like BEST (**Fig 9A**). This is despite the time taken to participate in BEST program activities. Similarly, faculty from both campuses who completed the Vanderbilt Survey believed BEST activities were valuable to both graduate (~80%) and postdoctoral (~60%) trainees (**Fig 9B**). Notably, a number of respondents (~40%) in the MSU survey did not know *at the time the survey* was taken whether the BEST trainees are benefiting from the BEST experience (*e*.*g*. response = neutral/don’t know). This was an essential factor to investigate given the concerns of faculty support going into this experiment.

### •Awareness of BEST as an institutional program and NIH Consortium

**Fig 10:**
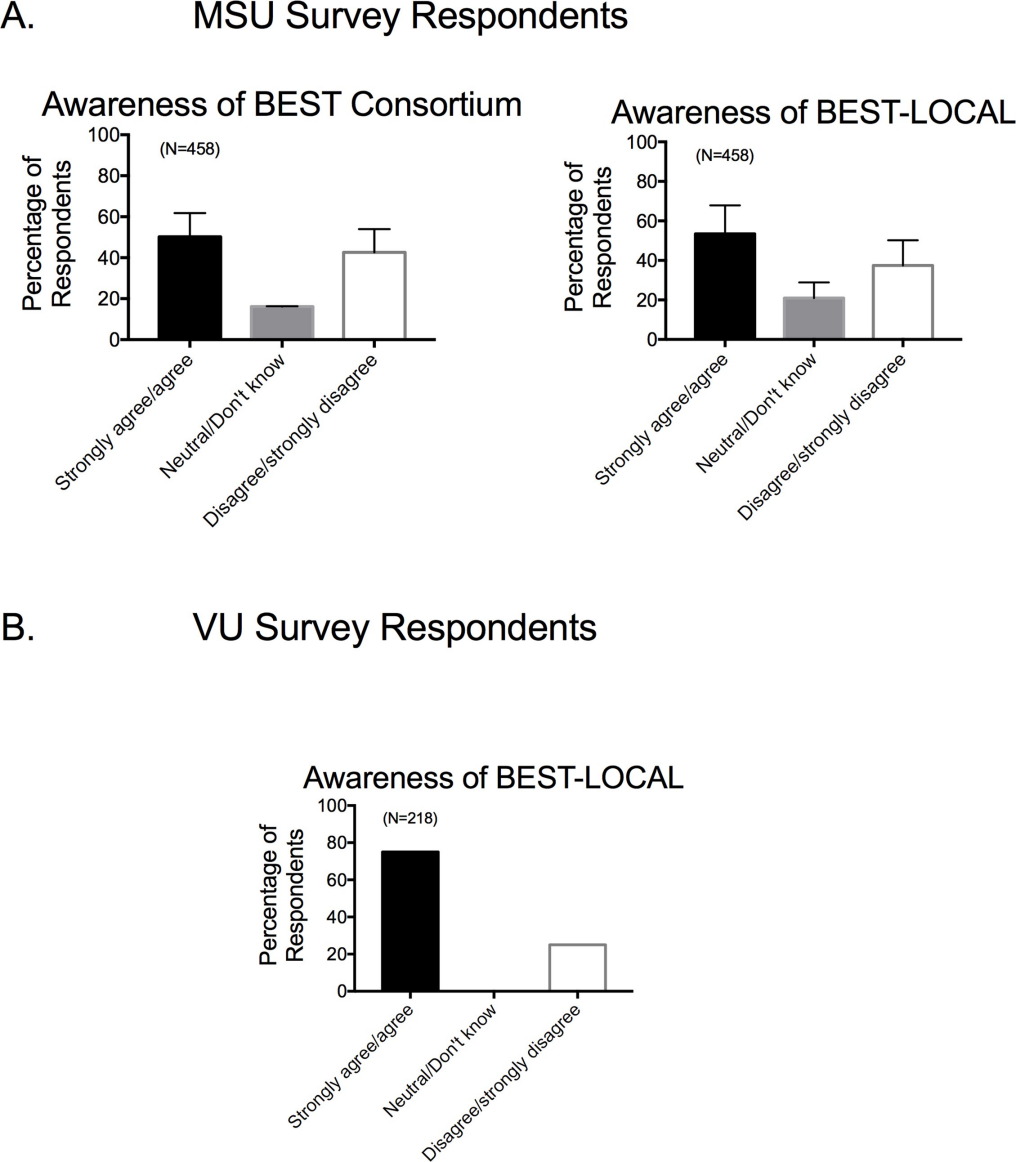
Responses to awareness of local BEST Programs and national NIH consortium for MSU (A) and VU (B) respondents. Bars are means±SEM for number of faculty indicated in parentheses.

A near majority of faculty taking the MSU based survey were aware that their institutions had a BEST NIH Consortium (**Fig 10A left**) and a similar percentage was aware of the BEST program on their campus (**Fig 10A right**). These data also indicated that, at the time the survey was taken, 50% of faculty at institutions with BEST Programs were unaware of their home programs and of the national BEST effort. Responses from the two institutions who participated in the Vanderbilt survey were different in that a greater percentage of faculty were aware of their local BEST program (**Fig 10B**).

### •Appropriate amount of time for trainees to be in career development training

**Fig 11:**
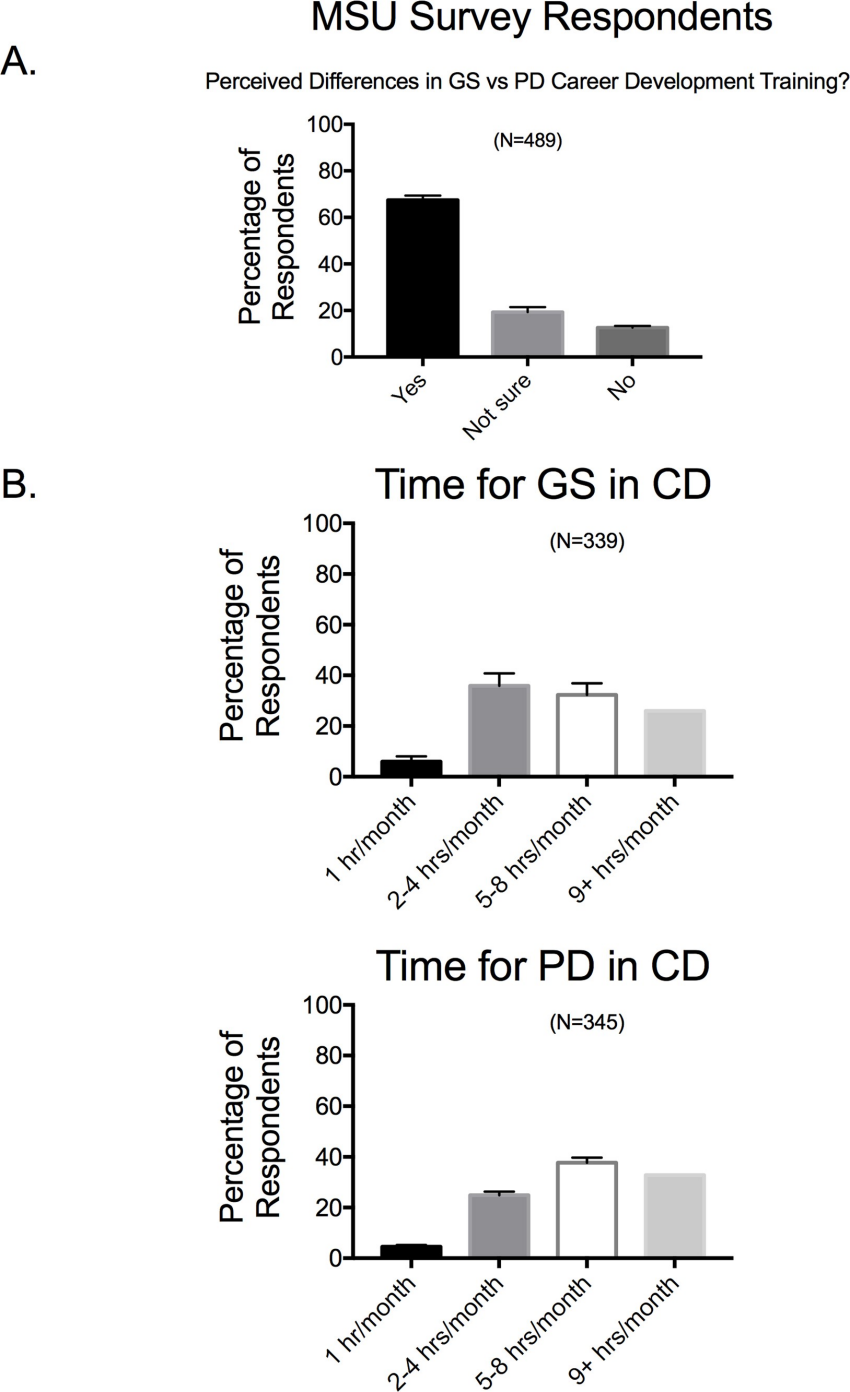
Responses to whether faculty perceive a difference in graduate *vs* postdoctoral fellow career development training (A) and reporting of hours suggested as appropriate (B). Bars are means±SEM for number of faculty indicated in parentheses. Reported for MSU based Survey respondents only.

Most faculty completing the MSU survey believe there is a difference in the training needs of postdoctoral fellows and graduate students (**Fig 11A**), but generally believed that post docs and graduate students should spend approximately the same amount of time on professional developmental activities. Faculty were asked the amount of time (hours/month) a graduate student or a postdoctoral fellow should spend on career development. Respondents were asked to bin these times into 1, 2–4, 5–8 or 9+ hours per month, and **Fig 11** shares these results for graduate trainees (**Fig 11B**) and postdoctoral fellows (**Fig 11C**). Statistically, these time allocations were not significantly different between the graduate and postdoctoral fellows. A number of respondents gave non-numerical answers to these questions, some of which are shared in **Table 2.** There were similar themes in these statements on career development for graduate students and postdoctoral fellows. These included the ideas that: 1) the answer depended on the stage of the student/fellow; 2) that both groups of trainees were already doing 100% career training: 3) what they did and the time they took to do it depended on individual goals.

**Table 2:** Selected non-quantitative answers to how much time a graduate student or postdoctoral fellow should spend in career development training.

**Graduate Student**
•This is difficult to answer if a student is supported 100% on the PI's grant and is expected to spend all time during research, and continued support of the grant depends on progress measured solely by publications
•This is a tricky question. Preparing for and giving talks, for example, is career development but is also part of their monthly routine as scientists. Assuming you mean time spent directly exploring job opportunities or in training outside of what is expected from being in a lab, I would guess 5 hours per month
•Depends a lot on the stage/phase of the work. Early, focus should be on getting an excellent piece of work accomplished. Toward graduation, last year or so, career development should be prioritized; probably to the tune of 3–6 monthly (I don't know what is available out there, 6 hours/month during one year may result in a lot of redundancy)
• From my perspective, 100% of what a graduate student does develops their career.
•It depends on individual.
•I have no idea
**Postdoctoral fellow**
**•** Depends on where they are in their position, early or late, and what they want to do.
**•** That depends upon what their goals are. Presumably, if they have selected top be a post-doctoral fellow, they are interested in a research based career at the outset of their studies. That may change as time progresses
**•** Strongly dependent on career stage, and the individual postdoc
**•** 100% since they are doing research
•I have no idea

Some faculty participating in the MSU Survey group were asked to provide a numerical response about the time they thought could be acceptably spent by a trainee in career development activities. Faculty (N = 146) answered 9.15±0.80 hours/month for graduate students *v*. 11.23±0.80 for postdoctoral fellows, values statistically different from one another (p<0.05). Thus, there is some evidence that supports that faculty believe career development training should be greater for postdoctoral fellows than for graduate students.

**Fig 12:**
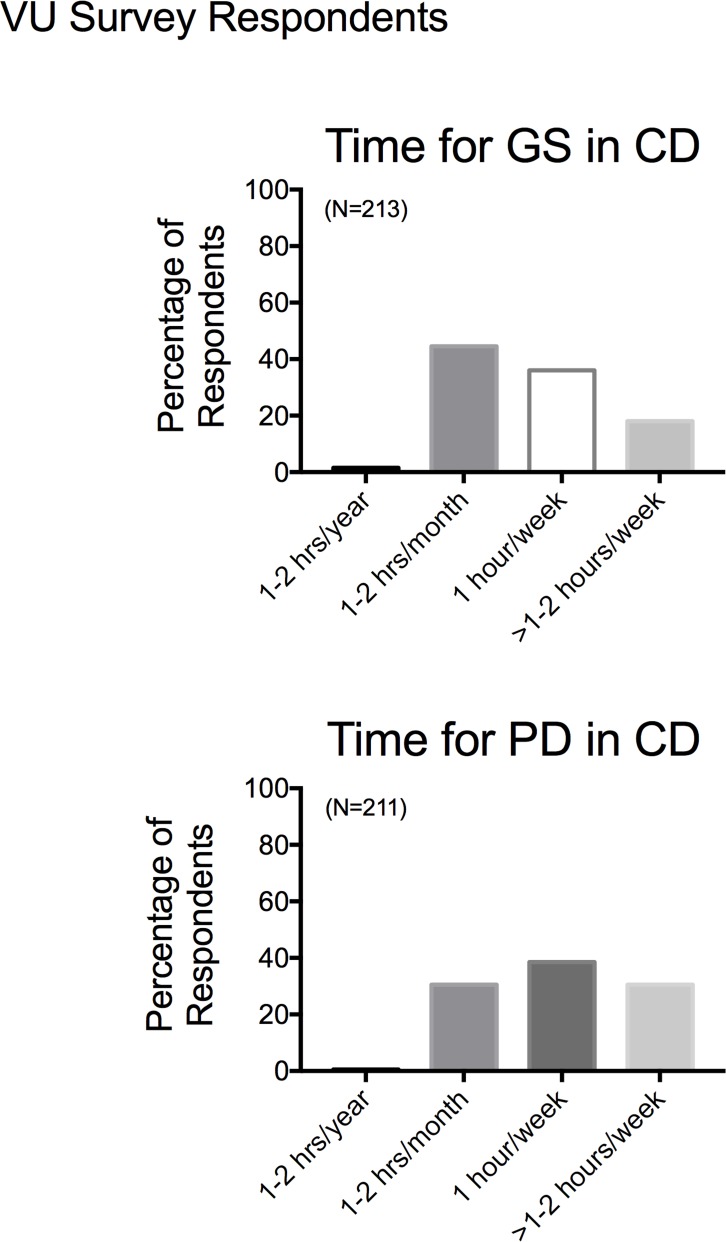
Responses as to perceived amount of time for graduate (A) vs postdoctoral fellows (B) to spend in career development training. Bars are means±SEM for number of faculty indicated in parentheses. Reported for VU based survey respondents only.

In a different approach, faculty participating in the Vanderbilt Survey were asked to choose what they thought was an appropriate amount of time to spend in career development activities for graduate students (**Fig 12A**) and postdoctoral trainees (**Fig 12B**). As was observed in the respondents from the MSU Survey, the range was wide. However, the collective findings support the faculty belief that postdoctoral trainees should spend a greater amount of time in their career development than graduate students. This could potentially indicate that faculty take it for granted that students—many but not all- pursue postdoctoral training after graduate studies and thus would be recipients of more focused career development training at such a time.

### •Institutional support for career development training

**Fig 13:**
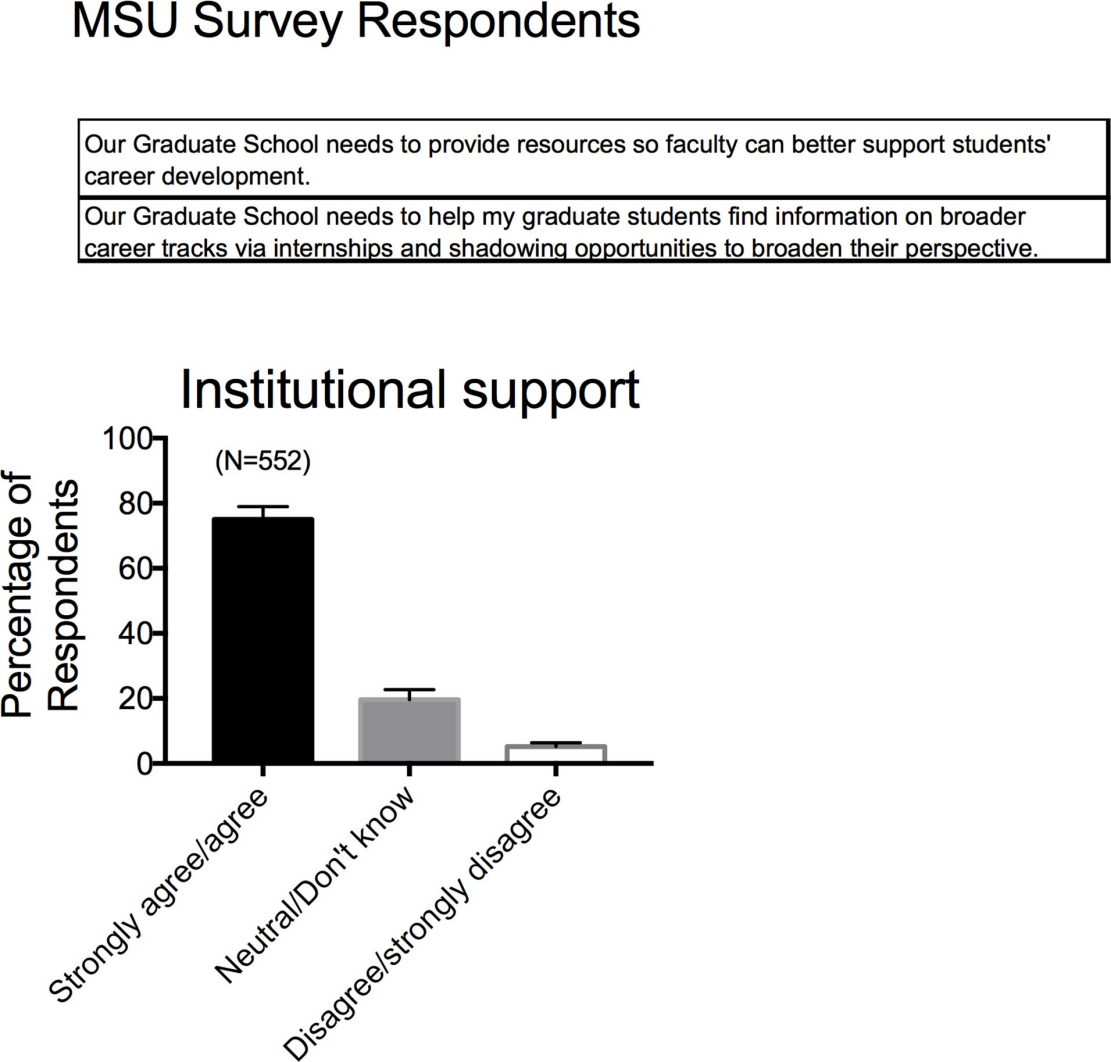
Responses to survey factor of institutional support for career development from MSU based survey respondents. Survey factor questions for MSU based survey are shown in the top of the panel. Bars are means±SEM for number of faculty indicated in parentheses. Reported for MSU based Survey respondents only.

**Fig 14:**
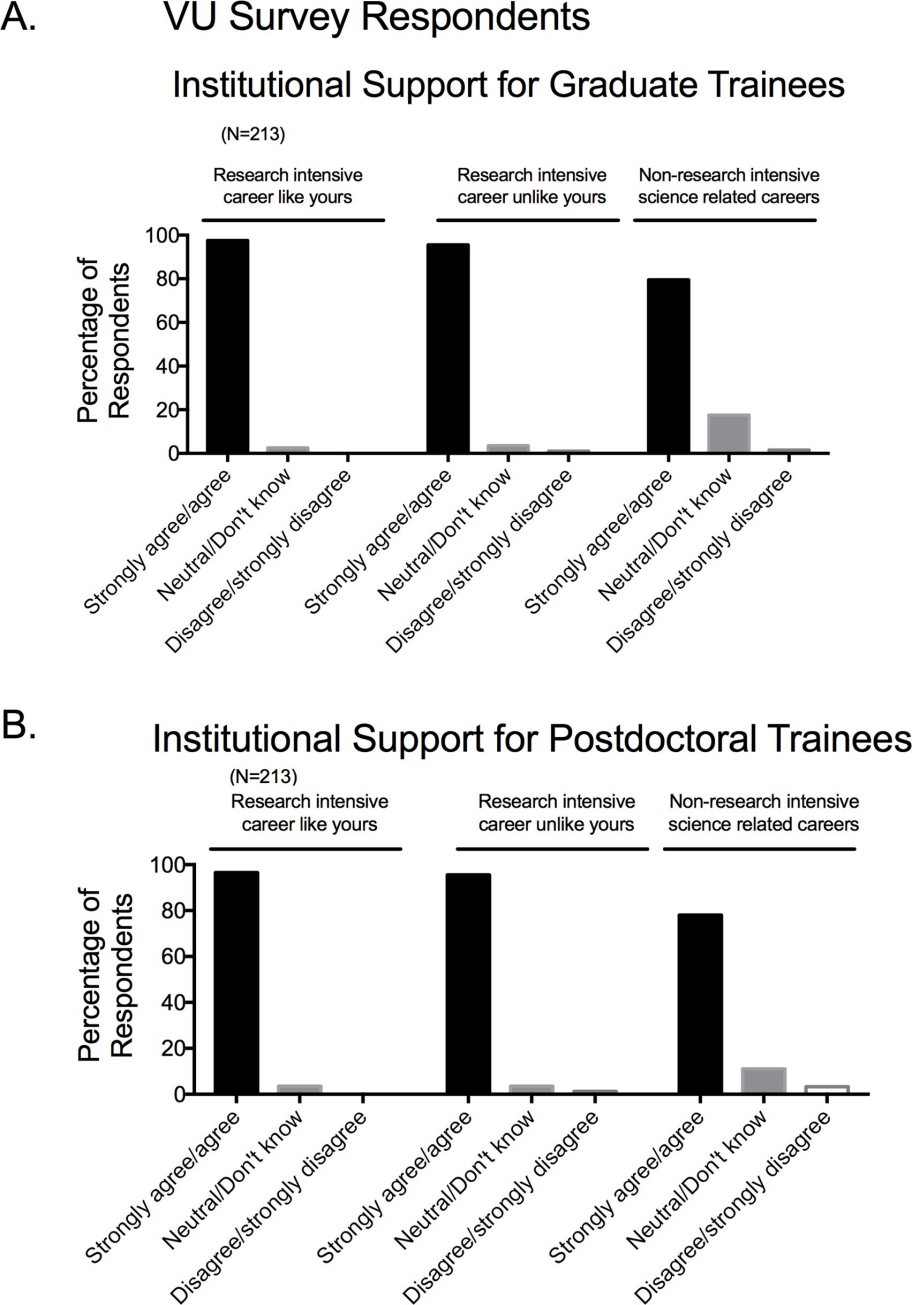
Responses to survey factor of perceived Institutional support for different biomedical careers for graduate (A) and postdoctoral (B) trainees for Vanderbilt based survey respondents. Bars are means±SEM for number of faculty indicated in parentheses. Reported for VU based survey respondents only.

Over 75% of faculty from the 5 institutions that took the MSU Survey believe that their institution–and more specifically their graduate school/division–needs to provide resources so that faculty can better support their trainees for a variety of careers (**Fig 13**). In particular, faculty report wanting support and resources that include helping to find and procure internships and shadowing opportunities. The Vanderbilt Survey posed this idea somewhat differently, asking whether an institution/graduate school/graduate division should support programs that prepare graduate students (**Fig 14A**) and postdoctoral trainees (**Fig 14B**) for: research intensive careers like that of the faculty taking the survey; research intensive careers that are unlike faculty positions; or non-research-intensive-but-science-related careers. These data strongly support the idea that faculty believe an institution should provide training for all science-based careers.

**Fig 15:**
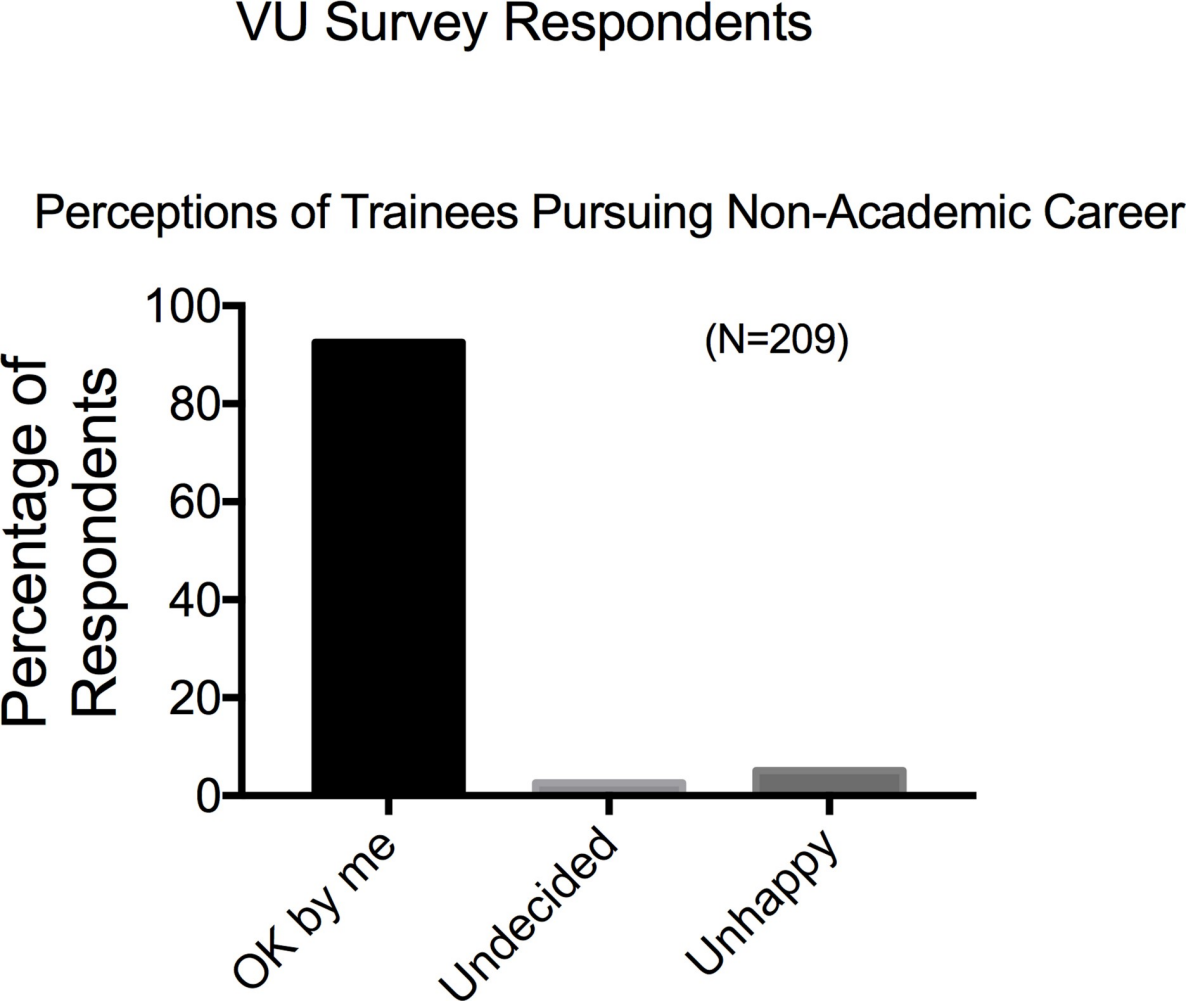
Responses to the question “Today, what is your evaluation of PhD students or postdocs in your lab who choose not to pursue a traditional academic faculty careers” to Vanderbilt based survey respondents. Bars are means±SEM for number of faculty indicated in parentheses. Reported for VU based survey respondents only.

The last piece of data, asked solely by the Vanderbilt Survey, was regarding the perception/feeling of faculty about their trainees pursuing non-academic careers. Overwhelmingly, faculty stated that such training was acceptable to them (**Fig 15**).

## Discussion

The intent of these surveys was to procure data about what faculty believe they know about career development training needs for biomedical science trainees, and to clarify their potential role in training students and postdocs in this arena. Faculty are such an integral part of biomedical training. The biomedical sciences have long adopted the apprenticeship model in which faculty are placed squarely in charge of such training. As such, their perception about what biomedical trainees currently need in terms of career training, and how they (the faculty) understand the options available to emerging biomedical scientists and engineers, is important not only to institutions with BEST Programs in place, but to all universities grappling with how best to prepare 21^st^ Century graduate students and post docs for the realities of the career landscape.

The BEST consortium is composed of seventeen institutions with different graduate education infrastructures and modes of student support. As a result, having two different surveys allowed us to capture the diversity between BEST programs and provide a rigorous approach by developing and performing the surveys independently. Before initiation of the BEST Programs and administration of these surveys, there was the notion that faculty might not be supportive of BEST programming because they, the faculty, viewed the primary goal of their training of the student to be discipline-based, and towards a career in academia. The clear conclusion from the present analysis is that the large majority of the faculty are sympathetic to and indeed supportive of vigorous career training for a wide range of careers that include faculty positions.

### Faculty embody the future

Most of the faculty surveyed report having trained a biomedical graduate student or post-doctoral candidate in the last 5 years, but most faculty have not had a trainee involved in a BEST program or BEST program opportunities. Faculty themselves reported having spent significant time on their own training, with the greatest percentage spending between 5 to < 8 years in graduate training, and anywhere from 0-<8 years in postdoctoral training. It is important to note that zero years–or no postdoctoral training period- was stated by some respondents. This is notable given that a question being asked within BEST and the scientific community as a whole is whether a postdoctoral fellowship is necessary for all career endeavors. Most interestingly, a high percentage (34.3±3.2%) of faculty had spent time being employed outside of academia; it was not asked whether this occurred during a break in employment from academia. Because of such experience, some faculty may have a greater appreciation for the breadth of possibilities available to a trainee as well as knowledge for the skills non-academic employers would hope trainees to have.

### Faculty understand the urgency of the problem

In both the MSU and Vanderbilt survey, faculty report an awareness that trainees were most likely not going to end up in an R1 institution in a research -intensive career such as their own. As such, faculty largely seem to recognize that we must better prepare our trainees for all careers. This observation is exactly where the value of BEST—and other programs like it—becomes clear: there is a need to enhance the doctoral experience to meet the developing needs for our knowledge-driven economies [6].

Faculty also report that they are having conversations about all careers with their trainees, at least to some degree. The Vanderbilt Survey asked, “Today, what is your evaluation of PhD students or postdocs in your lab who choose not to pursue a traditional academic faculty career?” Faculty taking this survey responded with a resounding “ok by me”. hat said, Gibbs et al [7] reported in 2015 that less than 1/3 of postdoctoral fellows receive career development from the department of which they are a part. These are important data to consider given the concern of present knowledge on demographics, career aspirations and outcomes of postdoctoral fellows within the US [8]. Future research should interview faculty members who have been identified by trainees as strong mentors for BEST, explore what makes them good mentors and how they prepare their students and ideally have these good practices be shared.

### Faculty have varied assessments as to the skills required for non-academic employees and would appreciate support

While faculty generally recognize that trainees need to develop skills that are applicable to careers inside and outside of academia, they had mixed opinions as to whether they were confident in their own knowledge of these skills, and the ability to keep trainees informed about fields different from academe. Regardless, they strongly believed that career development-related mentoring was a part of their job. It is here that career development programs and BEST-like activities are so crucial. Such programs provide career development resources not only to trainees but also to *faculty* who want to improve their own knowledge of career development. A testament to this finding is that over 80% of faculty surveyed—both in the MSU and Vanderbilt Surveys—believe that institutions should be training towards all types of biomedically related careers (research intensive and non-research intensive).

A goal of the national BEST consortium website (nihbest.org) is to share those tools that have proven themselves to be most useful, to encourage other institutions to embrace and adopt them so that career development training is more tenable, and to disseminate information through this website. Presently, this effort is paralleled by important work done by the National Research Mentoring Network [9] which focuses on mentorship and professional development, with an emphasis on diversity, inclusivity and culture. Importantly, trainees who have low perceived program support, and are particularly interested in careers outside academia, are less effective in their career searching [10]. This outcome suggests that explicit, solid support provided by faculty members, departments or institutions for broad career interests, would improve trainees’ ability to search for information and opportunities in careers of all kinds. Such career support could come in many different forms, including developing a system of trained career coaches such as those that have proven to be successful in supporting underrepresented minorities in the biomedical sciences [11]. This also includes PhD Career Services offices, which were in place in some but not all BEST institutions when the grants began. While such a Career Service Office is traditionally focused on the undergraduate population, having an office dedicated to the PhD and postdoctoral fellow is a form of career support.

### Faculty perceive a difference in what PDs vs GSs need in career development

Collectively, the responses from both the MSU and Vanderbilt Surveys reveal the faculty opinion that career development training should take at least the same if not more time for postdoctoral fellows *vs* graduate students. This outcome means, though, that faculty themselves have to support such an endeavor. In our surveys, “career development” was self-defined by the faculty, and BEST Programs have defined it broadly. Career development can include a variety of activities such as a course on “Business and Management Principles for Scientists” [12], professional mentoring by career counselors, workshops on different professional skills, and many other activities which are currently listed on the NIH BEST website. Selected non-numerical answers presented in Table 2 suggest that faculty think in pointedly different ways about what counts as career development, with some believing that everything a trainee does is career development to some having no idea what career development entails, time wise. Moreover, anecdoctal/theoretical concerns were raised that the needs of a trainee in career development are not believed to be constant, and were considered by some as highly individual, making it difficult to pinpoint a single appropriate time to start career development.

### Faculty perceived BEST Programs as beneficial to their trainees

The BEST Program was considered beneficial to trainees in a number of different ways. This included a feeling of general benefit, that students weren’t being delayed in their degree completion, that they were happier, that they were having a positive impact in the lab and had more direction in their own career development. These particular elements were key for BEST experimenters to investigate given concerns that BEST would extend time-to-degree, and reduce commitment to lab work and disciplinary productivity. Our finding supports the hypothesis that the resources provided within BEST are beneficial to the faculty in that they perceive a gap in their own and in trainee skills and knowledge surrounding career development, and this gap is filled by BEST Programs. The BEST Programs, at least in part, fulfill their mission in achieving buy-in amongst faculty. It would be ideal to share and spread the ideas from this work.

### Faculty vary in their knowledge about Local and National BEST Programs

The finding that faculty respondents across survey sites indicate that they are generally unsure about whether their colleagues discuss career options with their trainees is consistent with the finding that some faculty, at least as reflected with those that took the MSU Survey, are still not aware of BEST Programs and the NIH BEST Consortium. This was different for the two institutions that administered the Vanderbilt survey. This lack of awareness potentially suggests that career development concerns and solutions for them are not being discussed between colleagues and within departments as we would hope. Additionally, Vanderbilt is the home institution of BEST, administering the consortium. Moreover, both schools administering their survey began in 2013, and thus a BEST program was on their campuses for the longest time possible. Communication amongst these two groups would be one way to better communicate the existence and utility of BEST Programs. Enhancing awareness of these programs becomes the duty of the consortium. This could be fulfilled by the BEST consortium being more active in sharing experiments and experiences, sharing the programs developed, and encouraging other biomedical programs to visit *nihbest*.*org* and use developed materials that are freely available resources.

### Limitations

Our findings have several limitations. First, the results of two separate surveys—developed by different individuals and with non-identical questions–were pulled together. The challenges of doing this–having slightly different takes on questions that led to separate presentation of data–were outweighed by the benefits. This work is beneficial *because* it took two separate approaches and arrived at largely similar conclusions. A goal of the seven participating institutions was to work on behalf of BEST together, and this exercise allowed us to do so. Second, we acknowledge potential survey bias. Faculty voluntarily answered this survey, and those who responded are most likely to already be invested in the work of career development for biomedical scientists. This would add a positive bias towards our outcomes. Second, surveys were administered at different times during the BEST experiment, and issued only once. It is a plan to issue these surveys again after several years of the BEST Programs being integrated into the campus culture to determine if there is a shift in culture/perspective. Does career development knowledge and training permeate and become woven into all levels of a trainee’s experience, from a lab group to a colleague’s group to a department and The Graduate School/Graduate Division? Finally, even though a two-year difference existed between administration of the Vanderbilt Survey and the MSU survey, the qualitative outcomes are the same–they reinforce one another.

### How do we move forward?

•The NIH BEST Consortium has the responsibility to make biomedical faculty more aware of BEST Programs and to share career development tools for biomedical trainees. The fact that 50% of faculty at some of the institutions who took the survey did not know about their local BEST or the NIH consortium means that we have not transmitted our message effectively.

•Ideally, the Consortium needs to connect NIH BEST resources with other groups committed to career development of biomedical trainees. This is especially true given the newly released report by The National Academies of Sciences, Engineering and Medicine entitled “Graduate STEM education for the 21^st^ century” [13] in which a student-centered education, requiring development of true core competencies, empowerment of students and transparency in future work paths are just a few of the suggestions highlighted [14]. The NIH funded BEST program is harmonious with the findings of this report.

•Future research should also evaluate what type of activities the graduate schools could initiate that help students to broaden, and this is one of the explicitly desired outcomes of the National BEST consortium. For example, it is presently unclear what set of activities such as workshops, internships, colloquium, job shadowing etcetera best help to broaden students’ career exploration the most. It would also be critical to explore where and when in the process might we bring in faculty to support these interventions so they too can learn more about broadening careers and become a source of leverage for students to rely upon.

## Supporting information

S1 File MSU Survey. Survey items were gather into factors in which each of the questions were weighted equally to provide a final outcome for that factor. Factors/scales are in **BOLD**. Other prefatory questions concerning faculty rank, gender, years spent in graduate training, years spent in postdoctoral training (including 0 as a possible answer), employment outside of academe, appropriate time for graduate student to spend in career development training, appropriate time for postdoctoral fellows to spend in career development training and whether there is a perceived difference in graduate vs postdoctoral fellow needs in career development training were questions asked individually and with no grouping.
**1**. **Sense of Urgency**
*There is a shortage of tenure track positions in research universities in biomedical fields
*Potential Answers*: *Strongly Agree; Agree; Neutral; Disagree; Strongly Disagree*
**2**. **Need for Change**
*We need to better prepare biomedical graduate students (GS) and PDs for a variety of careers.
*Potential Answers*: *Strongly Agree; Agree; Neutral; Disagree; Strongly Disagree*
**3**. **Faculty Knowledge base**
•Generally speaking, I have a good knowledge base of skills non-academic employers require of GS/PDs.
•I think my faculty colleagues have a good knowledge base of skills non- academic employers require of GS/PDs.
*Potential Answers*: *Strongly Agree; Agree; Neutral; Disagree; Strongly Disagree*
**4**. **Student Career Knowledge base**
•I believe GS and PDs have a good knowledge base of skills non-academic employers may require of them.
•GS and PDs are well prepared to thrive in the everchanging biomedical workplace beyond a research based academic job. (3; Reverse-coded)
*Potential Answers*: *Strongly Agree; Agree; Neutral; Disagree; Strongly Disagree*
**5**. **Mentoring**
•I keep GS and PDs informed about job opportunities in nonacademic fields.
•I share personal experiences with GS and PD on career choices beyond academia.
*Potential Answers*: *Strongly Agree; Agree; Neutral; Disagree; Strongly Disagree*
• I provide GS and PDs with personal contacts/networking options with biomedical professionals to talk to the students about non-academic careers.
*Potential Answers*: *Strongly Agree; Agree; Neutral; Disagree; Strongly Disagree*
**6**. **Perceptions of Department Support**
•My department encourages GS and PDs to discuss nonacademic career options.
•My department provides resources to help GS/PDs understand non- academic options for careers.
•My department encourages students to broaden their experiences through shadowing experts or doing an internship in order to learn about career opportunities.
•My department encourages GS and PDs to discuss academic career options.
•My department provides resources to help GS/PDs understand options for academic careers.
*Potential Answers*: *Strongly Agree; Agree; Neutral; Disagree; Strongly Disagree*
**7**. **Perceptions of Colleagues’ Support**
•My colleagues encourage GS and PDs to discuss nonacademic career options.
•My colleagues provide resources to help GS/PDs understand non- academic options for careers.
•My colleagues encourage students to broaden their experiences through shadowing experts or doing an internship in order to learn about career opportunities.
•My colleagues encourage GS and PDs to discuss academic career options.
My colleagues provide resources to help GS/PDs understand options for academic •careers.
*Potential Answers*: *Strongly Agree; Agree; Neutral; Disagree; Strongly Disagree*
**8**. **Perceptions of Faculty Support**
•I encourage GS/PDs to discuss non-academic career options.
•I provide resources to help GS/PDs understand non- academic options for careers.
•I encourage students to broaden their experiences through shadowing experts or doing an internship in order to learn about career opportunities.
•I encourage GS and PDs to discuss academic career options.
•I provide resources to help GS/PDs understand options for academic careers.
•I am interested in enhancing my mentoring skills.
•I am concerned that time spent by my GS/PDs on internships and other career broadening activities will negatively impact lab performance.
*Potential Answers*: *Strongly Agree; Agree; Neutral; Disagree; Strongly Disagree*
**9**. **Perceptions of Graduate School Support**
•Our Graduate School needs to provide resources so faculty can better support students' career development.
•Our Graduate School needs to help my graduate students find information on broader career tracks via internships and shadowing opportunities to broaden their perspective.
*Potential Answers*: *Strongly Agree; Agree; Neutral; Disagree; Strongly Disagree*
**10**. **Awareness about BEST program at Home Institution and NIH Consortium**
•I am aware of NIH BEST Consortium.
•I am aware that an NIH BEST program is on our campus.
*Potential Answers*: *Strongly Agree; Agree; Neutral; Disagree; Strongly Disagree*
**11**. **If faculty Mentored a BEST student.**
•Are making timely progress on their degree completion. (32)
•Are happier for participating in the BEST program. (33)
•Have a positive impact on my lab.
•Have benefited from BEST (e.g. providing ideas, reducing worry) relative to career development.
*Potential Answers*: *Strongly Agree; Agree; Neutral; Disagree; Strongly Disagree*
(XLSX)

S2 File Vanderbilt University survey. * Questions were answered but are not included in the results of this present study.
1. **Prior to taking this survey, were you aware of your institution's BEST program?**
*Potential Answers*: *Yes*, *I was aware of my institution's BEST program and what it does*.
*Yes*, *I was aware but I didn't know much about my institution's BEST program; No*
2. **Have any of your PhD students participated in any part of your institution’s BEST program?**
*Potential Answers*: *Yes*, *at least one of the students in my lab has participated in at least one BEST program activity*.
*No*, *none of the students in my lab has participated in a BEST program activity*.
*I don't know if any of the students in my lab has participated in a BEST program activity*.
**3**. **What do you think is an appropriate amount of time for a typical PhD student to spend on his/her own career development?**
*Potential Answers*; *1–2 hours a year; 1–2 hours a month; 1 hour a week; more than 1–2 hours a week*
**4**. **For the PhD student(s) who participated in your institution’s BEST program, do you think that it was beneficial for him/her?**
*Potential Answers*; *Yes; No; I don't know*
**5**. Have any of your postdocs participated in any part of your institution’s * BEST program?
*Potential Answers*: *Yes*, *at least one of the postdocs in my lab has participated in at least one BEST program activity*.
*No*, *none of the postdocs in my lab has participated in a BEST program activity*.
*I don't know if any of the postdocs in my lab has participated in a BEST program activity*.
**6**. **What do you think is an appropriate amount of time for a typical postdoc to spend on his/her own career development?**
*Potential Answers*; *1–2 hours a year; 1–2 hours a month; 1 hour a week; more than 1–2 hours a week*
**7**. **For the postdoc(s) who participated in your institution’s BEST program, do you think that it was beneficial for him/her?**
*Potential Answers*; *Yes; No; I don't know*
**8**. **Do you think that an institution should help students prepare for: an academic career; a non-academic career**
*Potential Answers*: *Strongly Agree; Agree; Neutral; Disagree; Strongly Disagree*
**9**. **Do you think that an institution should help postdocs prepare for: an academic career a non-academic career**
*Potential Answers*: *Strongly Agree; Agree; Neutral; Disagree; Strongly Disagree*
*10. Three years ago, did you think that the majority of the following were interested in a tenure-track faculty position at an R1 institution?
*Potential Answers*; *Yes; No; I don't know*
*11. Three years ago, some people believed that students who did not go to a tenure-track faculty position inan R1 institution were underachieving. Did you agree with this belief?
*Potential Answers*: *Agree Completely; Agree Somewhat; Disagree Somewhat; Disagree Completely*
*12. Three years ago, some students and postdocs thinking about a non-academic career path said they did not want to talk about this issue with their research advisors.
*13. Three years ago, did you think that a student/postdoc highly interested in a non-academic career would be less productive in terms of research publications during their training in your lab?
*Potential answers*: *Yes*, *I thought these trainees were less likely to be productive in the lab; No*, *I did not think this was an issue*. *I expected the same degree of productivity regardless of a trainee's future career plans*.
*14. Three years ago, what was your evaluation of students or postdocs in your lab who chose NOT to pursue a traditional academic faculty career?
*Potential Answers*: *It was OK with me; I was on the fence; I was not happy with this*
**15**. **Today, do you think that the majority of the following are interested in a tenure-track faculty position in an R1 institution?**
*Potential Answers*: *Yes No I don't know*
**16**. **Do the following talk with you about non-academic career paths? your current PhD students your current postdocs**
*Potential Answers*: *Yes*, *frequently Yes*, *a little No*, *never*
*17. **Today, do you think that a student/postdoc highly interested in a non-academic career would be less productive in terms of research publications during their training in your lab?**
*Potential answers*: *Yes*, *I think these trainees are less likely to be productive in the lab*.
*No*, *I do not think this is an issue*. *I expect the same degree of productivity regardless of a trainee's future career plans*.
**18**. **In your opinion, what percentage of current students at your institution are likely to eventually obtain a tenure-track faculty position in an R1 institution?**
*Potential Answers*: *less than 25%; 25–50%; over 50%*
**19**. **Today, what is your evaluation of students or postdocs in your lab who choose NOT to pursue a traditional academic faculty career?**
*Potential Answers*; *It’s OK with me; I’m on the fence I’m not happy with this*
(XLSX)
